# The effects of bortezomib on the ovariectomy applied rat uterus: A histopathological, stereological, and immunohistochemical study

**DOI:** 10.22038/IJBMS.2018.24756.6152

**Published:** 2018-11

**Authors:** Habib Khoshvaghti, Berrin Zuhal Altunkaynak

**Affiliations:** 1 Department of Histology and Embryology, Medical Faculty, Bülent Ecevit University, Zonguldak, Turkey; 2 Department of Histology and Embryology, Medical Faculty, İstanbul Okan University, İstanbul, Turkey

**Keywords:** Bortezomib, Microscopy, Ovariectomy, Rat, Stereology, Uterus

## Abstract

**Objective(s):**

In this study, potential protective effects of Bortezomib (Bort), as a proteasome inhibitor, were investigated on the uterus of ovariectomized rats by histological, morphometric and immunohistochemical methods.

**Materials and Methods:**

In this study, 18 *Sprague dawley* strain female rats (12 weeks old, 250-300 g body weight) were used. Animals in the control group (Cont, n=6) were not exposed to any treatment. Ovariectomy was performed on the experimental groups. They (n=12) were divided into ovariectomy (Ovt, n=6) and Bortezomib (Bort, n=6) subgroups. Twelve weeks later, the rats were perfused. Then, uterine tissues were removed and examined by morphometrical, and light and electron microscopy methods. In addition, immunoreactivity of nuclear factor-kappa (NF-κB) was evaluated.

**Results:**

Morphometric and histopathological evaluations showed that Bort was effective in the uterus and protects the layer structures and the cells.

**Conclusion:**

In the light of these findings, we suggest that for proteasome inhibitor particularly Bort is thought to be useful through proteasome inhibition and NF-κB pathway.

## Introduction

Biological aging is associated with gradual decrease of cell metabolism and basic functions; inevitably it is a process that resulted in death (1). According to many researches, mitochondrial dysfunction is thought as one of the main reason of aging (2). But, many researchers reported that the mitochondrial dysfunction during cellular aging occurs independently from other stimuli such as telomere dysfunction, oncogene activation and genotoxic stress (2-4). The changes in lysosomes of senescent cells and tissues are among the most commonly observed aging indicators, and they are used as the aging indexes in literatures (5). Lysosome is responsible for the degradation of many macromolecules including proteins. Studies indicated general decline in peroxisomal function with aging (6). Although the number of peroxisome increases in aged rats, the size of peroxisome decreases (7). With aging, catalase and acyl-CoA oxidase contents are reduced in peroxisomes, whereas the thiolase and urate oxidase contents are increased (8). In addition, the disorganization of the nuclear structure is seriously associated with aging (9). 

Nuclear factor-kappa (NF-κB) is a transcription factor; it promotes some cytokines, cell adhesion molecules, transcription of pro-angiogenic molecules and suppresses apoptosis (10, 11). It is a specific factor in the cytoplasm of the unstimulated cells that inhibits gene transcription (11-14). We used NF-κB immunoreactivity as an indicator of the apoptotic response.

Ovariectomy is the surgical removal of the ovaries. Ovariectomy is an operation that may be performed in some diseases such as cancer or ovarian cysts or can be performed with prophylactic hysterectomy to reduce the chance of development of ovarian and breast cancer (15). However, this operation has serious long-term consequences due to hormonal factors (16). Women who have undergone bilateral ovariectomy have often lost their reproductive function and the ability to make the oestrogen and progesterone hormones, whereas in natural menopause, the production of hormones, especially low levels of androgens, continues in ovaries (17). The side effects and other risks of ovariectomy include premature death, cardiovascular diseases, cognitive disorders, dementia, Parkinsonism, osteoporosis, and bone fractures (18-20).

Both normal and the cancer cells contain proteasome. General duty of proteasomes includes degradation of damaged or useless proteins via running an enzyme that the procedure is named proteolysis. The primary function of the proteasome is to regularly degrade damaged proteins within the cell (21). Proteasome contributes directly or indirectly in many cellular processes like apoptosis and protein synthesis through recycling, the cell cycle, endocytosis, transcription, organelle biogenesis, spermatogenesis, and angiogenesis (22). When the degradation of protein is impaired, probably it results in the accelerated and uncontrolled mitosis, which develops to cancer (23). Proliferation and survival of cancer cell depends on the regulated proteasomal protein. Proteasome inhibitors unlike non-dividing cells, induce apoptosis in tumor cells (24-28).

Bortezomib (Bort) has emerged as a rational approach for treatment of cancer (29) and it is the first proteasome inhibitor for the treatment of multiple myeloma and mantle cell lymphoma that has been approved by the US Food and Drug Administration (FDA) (30). It inhibits proteasome enzyme complexes, as the initial treatment of the disease or is used in combination with various drugs. This deterioration occurs in the cascade of signals in cancer cells, and it may cause the inhibition of cell death and tumor growth (31, 32). Specifically, proteasome inhibitors cause apoptosis in cells that can be proliferate; therefore, it can be more effective in a more advantageous position than other anticancer drugs for treatment of cancer.

In the current study, to detect the possible protective effects of Bort, it was used as inhibitor on the uterus of the rats, which were subjected to ovariectomy procedure, and their uterus was investigated by histopathological and immunohistochemical methods. 

## Materials and Methods

***Experimental animals***

All experimental procedures were approved by the Institutional Committee on Animal Care, Use, and Research and performed according to the Guide for the Care and Use of Laboratory Animals (Experiments Local Ethics Board, No. 115-36643897 decision was made in accordance with the rules and ethical guidelines, Ataturk University, Turkey, revised 2014).

Eighteen 12-weeks-old female rats (*Sprague dawley*) were used. The materials were obtained from Atatürk University Medical Experimental Research and Application Center (ATADEM). Animals were divided into three groups (n=6 per group) as follows: Group I (Cont): Healthy and untreated Control group. This group was perfused after 12 weeks of feeding in the same laboratory conditions without any application of experimental procedures. Group II (Ovt): Animals in this group were perfused after 12 weeks and ovariectomy operation, at the age of 24 weeks. Group III (Bort): After waiting for a healing process of 8 weeks, the ovariectomized rats in this group were administered with 0.2 mg / kg Bort intraperitoneally (IP) during the period of 4 weeks, 2 times in a week.

All rats were anesthetized with ketamine (IP, 10 mg/ 100 g body weight; Sigma Chemical Comp., St. Louis, MO, USA) and xylazine (50 mg/ 100 g body weight; Sigma Chemical Comp., St. Louis, MO, USA.) at the end of the experiment. After perfusion, the uterus tissues of all rats were removed. Immediately after taking the uterus biopsies, they were fixed in 10% formalin solution for 72 hours. Then, the obtained tissue samples were evaluated by routine histologic, immunohistochemical and stereology methods.

**Figure 1:**
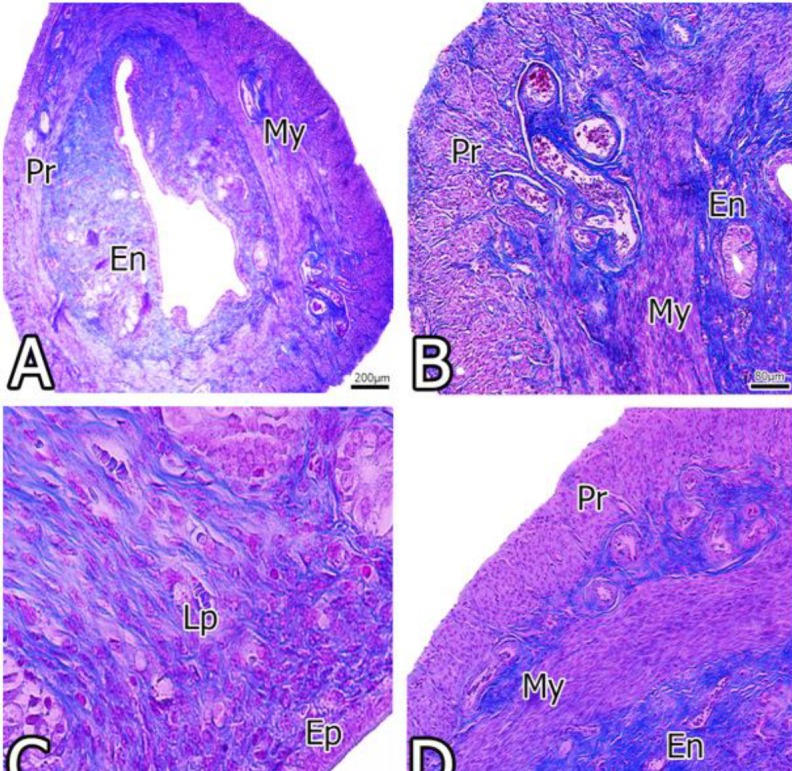
Images obtained from the uterus of the Cont group. Different sections of the endometrium, myometrium and perimetrium were in normal view. **En**, endometriyum; **My**, myometrium; **Pr**, perimetrium; **Ep**, epithelium; **Lp**, lamina propria

**Figure 2:**
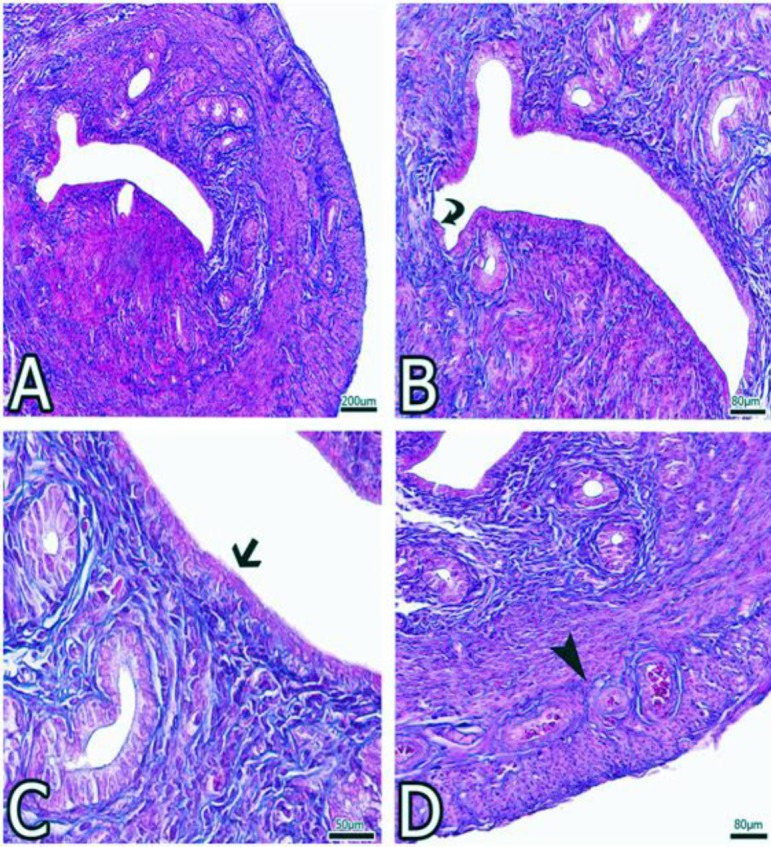
A-D indicate the images obtained from the uterus of the Ovt group’s. ♦, thinning of the epithelium; →, epithelial thinning; ► , proliferation in the blood vessels

**Figure 3:**
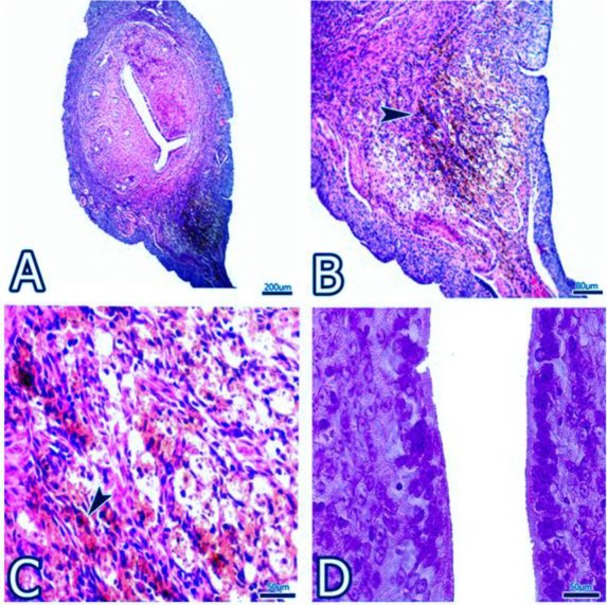
A-D show the images obtained from the uterus of the Bort group’s. ►, proliferation of myometrium muscle cells. In D part, epithelium and stroma also draws attention to the healthy appearance

**Figure 4:**
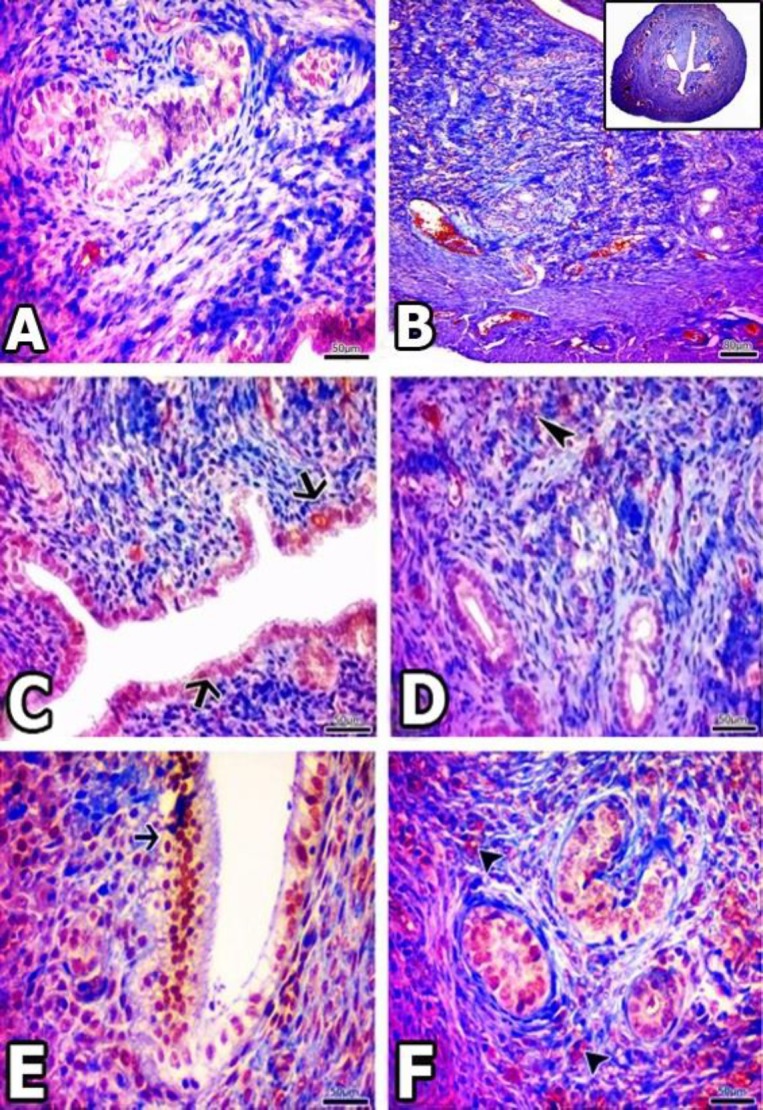
A and B are images obtained from the uterus of the Cont group. C and D are images obtained from the uterus of the Ovt group. In the C and D, in the endometrium, immune positivity was observed in connective tissue cells that forming the uterine stroma. → and ► show immune cells as epithelial and stromal positive staining. E and F are images obtained from the uterus of the Bort group. C shows intense cytoplasmic immune positivity in the stromal cells of the endometrium. D, capillary wall is observed positive immunostaining. → and ► respectively uterine epithelial surface and shows positive staining observed on the vessel wall. These representative images stained with anti-NF-kB immune peroxidase & Mayer hematoxylin.

**Figure 5:**
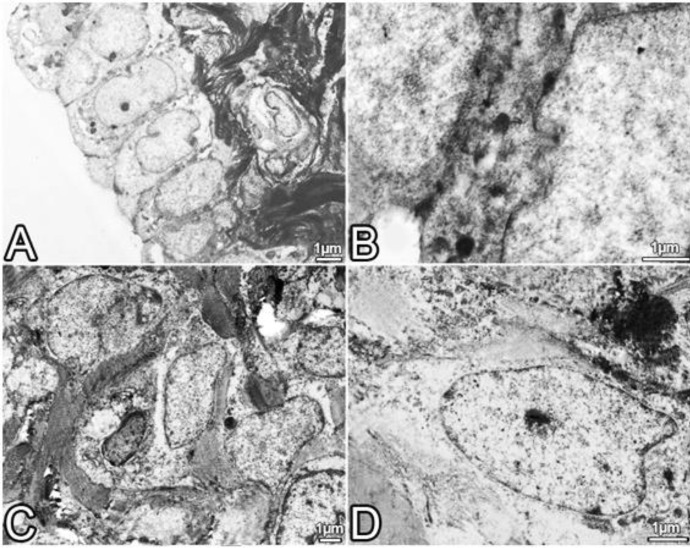
A-D are the electron microscopic images which belonging to the Cont group of uterus. A, endometrial surface epithelium; B, complex of side face connections between epithelial cells; C and D, stromal cells and connective tissue fibers are regarded as healthy

**Figure 6:**
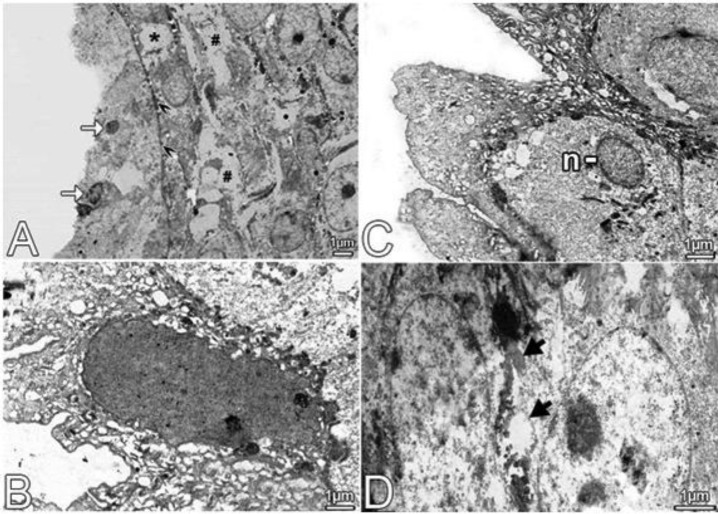
A-D are the electron microscopic images which belonging to the uterus of the Ovt group. In A-D, degeneration observed in surface epithelium of endometrial and the side face of complex connections between epithelial cells it is monitored in detail. →, Shrunken and pyknotic appearance until the nuclei of epithelial cells. *; vessels in the stroma; #, irregular limited spaces in the stroma;►, thickened basement membrane; n, pushed to the edge shrunken and epithelial cell nucleus; →, deteriorated between epithelial cells and electrons shows the intercellular junctional complexes containing dense material. B and C show electron-dense organelle structure and epithelial cells with shrunken nucleus.

**Figure 7:**
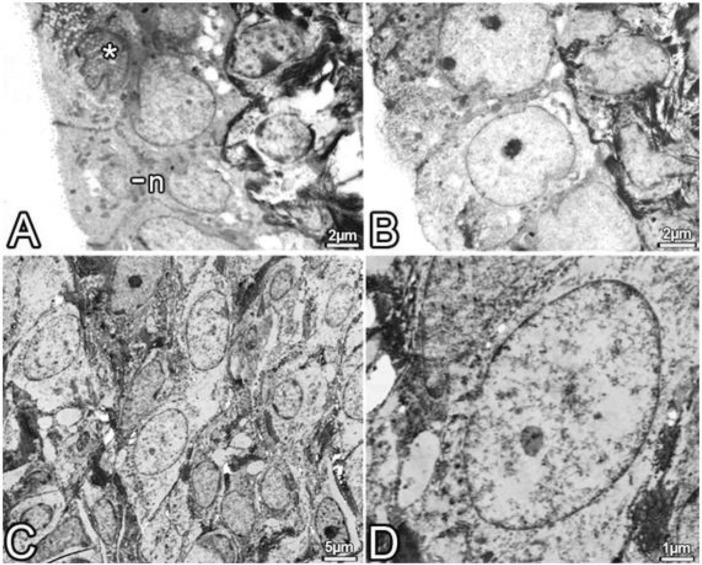
A-D notice that the electron microscopic images which belong to uterus of the Bort group. Surface epithelium of uterine and the endometrial stroma, it is mostly noteworthy healthy appearance. n, basal located nucleus of epithelial cell; *, intensive looking electrons, it shows prominent nuclei. B, intercellular connections, it draws attention to a healthy structure

**Table 1:** Morphometrical evaluations for each group (mean±SEM)

Estimation	Cont	Ovt	Bort
Endometrial thickness (mm)	1.523±0.072	0.664±0.106	1.066±0.191
Myometrium thickness (mm)	0.371±0.073	0.242±0.024	0.297±0.051
Perimetrium thickness (mm)	0.261±0.034	0.192±0.023	0.243±0.012
Endometrial volume (mm^3^)	273.93±21.90	85.61±13.75	175.49±19.69
Average uterine gland area (mm^2^)	0.015±0.0008	0.012±0.001	0.014±0.0012
Average uterine gland volume (mm^3^)	0.05 ±0.003	0.059±0.007	0.049±0.002
Myometrium volume (mm^3^)	203.76 ±16.93	73.54±15.02	124.16±11.88
Perimetrium volume (mm^3^)	230.06±34.63	119.25±25.57	183.73±29.48
Vascular volume (mm^3^)	78.89±17.57	23.24±12.70	18.57±4.12

***Ovariectomy operation***

The second and third group female rats were under anesthesia with 20 mg/kg dose thiopental sodium. After that, 2-3 cm incision, reaching the ovaries, was made in the lateral side of abdomen, and the ovaries were taken unilaterally. Metimazole sodium was provided postoperatively for anesthesia, with a dose of 25 mg/kg. For a week, infection was prevented by giving appropriate doses of penicillin for rats every day and making wound dressings. After being ovariectomized, the rats were fed with normal diet and water for 8 weeks to apply aging; it has been adjusted to the required time. Bort application procedure was started after 8 weeks (60 days) from ovariectomy operation. For this purpose, 3.5 mg Bort was dissolved in 7 ml isotonic solution and 1 ml of the solution containing 0.5 mg Bort was divided into falcon tubes. This solution of 8 ml was injected to each rat in the Bort group. In other words, each rat was administered with IP 0.2 mg/kg Bort. This application was repeated two times a week for 1 month.

The uteruses were then removed from all animals and processed by graded alcohols (Sigma Chemical Comp., St. Louis, MO) and xylene (Sigma Chemical Comp., St. Louis, MO). Then, they were immersed in paraffin series and embedded in fresh paraffin (Merck, Darmstadt, Germany).

Sections were cut into 5 μm thicknesses from the blocked uterus tissues by using a rotary microtome (Leica RM2125RT). Each slide was stained with hematoxylin–eosin dye (H–E) for light microscopic examination, and stereological analysis (Cavalieri Principle and Nucleator method). For stereological analysis, a computer-assisted analysis system called Stereo Investigator software version 9.0 was used (Micro Bright Field Inc., Colchester, VT).

***Immunohistochemical procedure***

For the immunohistochemical evaluation, sections were taken to polylysine glass and were then stained for NF-κB (p65) using streptavidin-biotin-peroxidase method (Rabbit polyclonal ab, Ab-1 p65, dilution 1.75). After the process of sections deparaffinization, to block endogenous peroxidase activity, they were prepared in distilled water and incubated for 30 min in 10% hydrogen peroxidase. For removal of antigen, sections were boiled in 10 mmol l^-1^ citrate buffer (pH 6.8) for 10 min at high set in microwave. Waited at room temperature for 20 min with Ultra V block, tissues preincubated dropwise with primary antibody for an hour. Then, it was incubated for 20 min with secondary antibody labelled with biotin and 30 min with streptavidin peroxidase complex. Chromogen 3-amino-9-ethyl carbazole and Mayer hematoxylin were used for staining the floor. Before dropping primary antibody, similar processes were also applied for negative control.

***Electron microscopy***

When animals under anesthesia, 5% glutaraldehyde were dropped on the uterus and then uterine tissues were removed. Then, it was incubated for 1 hr in 5% glutaraldehyde solution. At the end of incubation time, it was washed with Millonig buffer 4 times for 15 mi. After washing with buffer, it was incubated with 1% osmium tetroxide for 1.5 hours in the dark. It was washed again with Millonig buffer 4 times for 15 min and the dehydration process was passed. For dehydration, the tissues were incubated in acetone series, propylene oxide and Araldite CY 212 (Agar Scientific; İstanbul; Turkey), respectively. After polymerization, sections at 70 nm thickness were taken on the 200 mesh copper grids by an ultramicrotome (Leica Ultracut UCT, Leica Microsystems GmbH, Germany). Then, the grids were examined and photographed under the electron microscope (JEOL JSM-7001, Tokyo; Japan).

***Stereological analyses***

In this study, morphological structure of the uterus was evaluated stereologically. For this purpose, the thickness of the endometrium, myometrium, and perimetrium layers was detected. Besides the total uterine volume, lumen of uterine, layer of endometrium, myometrium, perimetrium and volume of vessels located in this layer was calculated with Cavalieri principle. Furthermore, the mean diameter and area of uterine glands were calculated by using the Nucleator method.

***Volume estimation with the Cavalieri principle***

The most commonly used method in stereological volume calculations is the Cavalieri principle. In this study, the volumes of sections taken from uterus samples were estimated using Cavalieri principle (33, 34). As for the Cavalieri principle, a data called coefficient of error (CE) is obtained to calculate the reliability of the point density of the grids and sectioning intervals (35). Previously published formulas were used to calculate the CE and coefficient of variation (CV) (36). Uterus volumes were calculated using the following formula (33).

 V=t x a/p x ΣP

Where V, is the mean volume of the uterus; t, the mean section thickness; a/p, the inter-point area; and ∑P, the total number of points hitting whole serial sections of the uterus.

***Estimating the average area of uterine gland with nucleator method***

The average area of the endometrial glands in all sections was calculated by computerized stereological analysis system. In this process, the basic principles of stereology “CE” were held in mind. Counting took place by considering the coefficent error value; after switching to other areas of respective field, randomly and step by step scanning the entire surface of a predetermined range of systematic section was started. After determining the appropriate step interval in the X-Y plane, range specified in this step area was used in the cross-sectional plane to perform measurements of uterine gland, and counting predetermined rules ‘counting frame’. At this point, average uterus gland area was estimated according to rules of counting frame and unbiased nucleator method.

The average area of the uterine glands in this study was estimated for calculating the following sequence:

1- Areas with the interested object (uterine glands) were determined on cross sections.

2- Interested area was drawn between lines and calculated in Stereo-Investigator program in Stereo-Investigator program.

3- Reference sectional area was multiplied by number of the steps that include equally spaced areas.

***Statistical analysis***

Data are presented as Mean±SEM, and were statistically analysed. Differences among groups were assessed by ANOVA with *post hoc* test to identify individual group differences. Differences were accepted statistically significant at *P*<0.05 level. 

## Results

***Stereological results***

 All morphometrical evaluations for each group (mean±SEM) are observed in Table 1. 

Compared to the Cont group, it was observed that the uterine endometrial thickness is significantly reduced in other groups (*P*<0.01). However, Bort group, in comparison with Ovt group, had significantly thicker endometrium (*P*<0.05). When subjects of Ovt group was compared to the Cont group, it was observed that the thickness of the myometrium significantly reduced (*P*<0.01). There was no significant changes between Count and Bort groups in terms of the myometrium thickness (*P*>0.05). In comparison with the Cont group, subjects of Ovt group displayed significant reduction in thickness of the perimetrium (*P*<0.05). Between Cont and Bort groups, in terms of perimetrium thickness, no significant changes were observed (*P*>0.05). 

***Volumetric results***

When Ovt group compared to the Cont group, there was significant reduction in endometrial volume (*P*<0.05). Bort group was found to have significantly increased volume of the endometrium in comparison with the Ovt group (*P*<0.05). Between Cont and Bort groups, no significant differences was observed (*P*>0.05). Compared to the Cont group, animals of Ovt group displayed significant reduction in mean uterine gland area (*P*<0.01). The mean area of the uterine gland significantly increased in Bort group compared to the Ovt group (*P*<0.05). No significant differences were observed between Cont and Bort groups in terms of the average area of the uterine gland (*P*>0.05). There were no significant differences among the groups in terms of the mean uterine gland volume (*P*>0.05). Also, a significant reduction was observed in myometrium volume of the Ovt and Bort groups compared to the Cont group (*P*<0.01). There was no significant difference between Bort and Cont group (*P*> 0.05) in terms of the myometrium volume. However, the volume of perimetrium was significantly increased in Bort group in comparison with the Ovt group (*P*<0.05). Volumetric values of perimetrium were observed to be significantly reduced in the Ovt and Bort groups in comparison with the Cont group (*P*<0.01). Between Ovt and Bort groups, there was no significant difference in terms of volumetric values of perimetrium (*P*>0.05).

***Histopathological results***

Findings showed that the endometrium, myometrium and perimetrium layers surrounding the centrally located lumen were found to be normal and healthy appearance in the Cont group. Simple columnar epithelium occupying the endometrial surface was observed as normal appearance (Figure 1).

In the Ovt group, uterus epithelium exchanged from the simple columnar to simple cuboidal form. Inflammatory cells were observed in the lamina propria located underneath the epithelium. In particular, there is a decrease in collagen fibers in the stroma, and proliferation was observed in stromal cells. Also, atrophy of endometrial glands and heterochromatic nuclei were observed as differences in the structure of cubic epithelial cells. In some areas, epithelium was observed in the squamous form. Proliferation of blood vessels was found in the stratum vasculare (Figure 2).

In the Bort group, the simple columnar epithelium lining of lumen was observed as mostly preserved. However, abnormalities in uterine glands were not observed. In particular, together with a decrease in the stromal connective tissue fibers, the normal frequency of stromal cells was noticed. Smooth muscle cells that form the structure of the myometrium were also observed more frequently in this group (Figure 3).

***Immunohistochemical results***

At the end of detailed examination for Cont group, few of the NF-κB positive cells were observed in endothelium and blood vessels between the stratum vasculare of myometrium and perimetrium.

When the immunologic activity of NF-κB (p65 sub-unit) was evaluated in the Ovt group, simple columnar epithelial cells of endometrium in uterine gland showed positive staining. Similarly, immune-positivity was observed in circular and longitudinal smooth muscle layers of myometrium. Immunoreactivity was observed in the stratum vasculare and a plurality of blood vessels in the stroma and also appeared heavily in endothelial cells. In addition, a strong NF-κB involvement was observed in the stromal cells. Usually, this cellular involvement is found in cytoplasmic level and in nucleus.

In the Bort group, NF-κB immunoreactivity was evident in epithelial and stromal cells. Positivity is observed with doughtily in cytoplasm of some stromal cells but weakly in nucleus. Immune-positivity was also observed in the gland epithelial cells and single layer epithelial of endometrium. In the vessels and between the muscle layers, significant immunoreactivity was detected. 

All immunohistochemical results are observed in the Figure 4.

***Electron microscopical results***

In the Cont group, endometrium was normal in terms of the fine structure. Apical cytoplasm of epithelial cells included polyribosomes. Cell surface and lateral connections were consistent with the structure of normal epithelium. There were not any abnormalities in stromal cells and the connective tissue fibers in the lamina propria.

In examination on uterine samples of Ovt group, a decrease of pinopodes in the surface epithelium in endometrium, the extension and the blunt, irregular apical cytoplasm was detected. In the cellular matrix, lateral and apical cell cytoplasm was more electron-dense in comparison with the Cont group, and an increase in the granular endoplasmic reticulum (GER) tubules was observed. However, there was an increase in the number of lysosomes. Junctional connections were observed by electron-dense content and borders to be joined were quite evident. Lamina propria was remarkable in irregular spaces. In stromal cells, some of the electrons in terms of both core and cell matrix were dense in appearance. The extracellular matrix between the stromal cells was poor compared to the Cont group in terms of connective tissue fibers. Connective tissue between the cells formed by fiber bundles was fine with electron-dense appearance.

In the Bort group, the structure of the endometrial surface epithelium was normal in view. Organelles such as lysosomes and mitochondria with healthy appearance were remarkable in the cell cytoplasm. Specializations of the apical face of epithelial cells were also regular and normal view. Rarely electron-dense and notched cells having nuclei, between healthy epithelial cells, were observed. Cells in the space between the connective tissue fibers were also monitored as well.

All electron microscopic results are shown in the Figures 5-7.

## Discussion

Bort prevents proteasome functions and is known as an agent with antitumor properties (29, 37, 38). In all cells in which proteolysis occurs by 26 S proteasome, it is considered as a compulsory metabolic process. In the cell cycle path, transcription of some proteins has roles in vital functions of cells and is responsible for the structure of proteins. Also, proteasome causes degradation of old, oxidized and the damaged proteins (37, 39, 40). The disruption of this order leads to cell apoptosis. After failures in normal functioning of proteins, ubiquitin / proteasome pathway is activated through proteasome inhibitors, and apoptosis is induced. Myc oncogene protein, cyclins, and regulatory molecules such as p53 are the most important elements involved in ubiquitin / proteasome pathway. Destruction of these molecules currupts the control of cell proliferation and cell cycle. 

Bort seems to affect on mitogen-activated protein (MAP) kinase signaling pathway and inhibits cell proliferation through caspase-dependent apoptosis and inhibition of NF-κB. Also, it appears to decrease the expression of adhesion molecules (41, 42).

Unsal and Sönmez (2014) conducted a study after ovariectomy of rats and showed that there is no histological changes in endometrium, and reported simple columnar epithelium, and myometrium. They also reported that thinning of the uterine wall was not observed in 5^th^-7^th^ days (43).

In the uterine cycle, changes in myometrium and endometrium are observed. There are receptors belonging to the sex hormones in smooth muscle cells of myometrium. But, after menopause, both the number and size of smooth muscle cells in the myometrium could be decreased due to lack of sex hormones (especially estrogen) (44). 

In the current study, the volume and thickness of the uterine layers were higher in the Cont group, but the minimum value was found in the Ovt group. According to findings of the present study, the thickness and volume of the uterine layers increased in Bort group. In the uterine gland area, the lowest values observed in the Ovt group and the highest values were obtained in the Bort group. Fawcett and Dean (1951) reported that atrophy was observed in uterus of ovariectomized rats (45). There was a difference that columnar epithelium was transformed into cubic epithelium and atrophy was observed in endometrial glands, fibers of the stromal connective tissue were tight and heavily regulated, and smooth muscle cells and stromal cells have been shown to be hyperchromatic (44). 

In aging, some changes such as thickening in the basal lamina of the epithelium can be observed in endometrial epithelium. In our study, epithelial cells of Ovt group had electron-dense and shrunken nuclei and organelles. In addition, the cells in the Ovt group had deterioration of the structure in the lateral surface connections. In this case, cells are considered as a part of the death process. In Ovt group, lamina propria stromal cells were sparse and they were not in healthy structure, whereas the amount of collagen fiber bundles was smaller and they looked as electron-dense structure. 

Steroid hormones and growth factors regulate the actual growth in the endometrium, in addition to differentiation and apoptotic events. Deterioration of the balance between cell proliferation and apoptosis, due to aging, can lead to many problems such as endometrial cancer. In the literatures, high apoptotic index has been reported in the endometrium of aged rats (46).

In the present study, we observed apoptotic cells in the Ovt group in endometrium, especially in the stroma.

According to the results of this study, it is thought that Bort may have protective effect in both histopathologic and morphometric views. When we compared the Ovt and Bort groups, it was noticed that the native structure of the epithelium was maintained in Bort group. Among the effects of aging at the cellular level, NF-κB induction is claimed to be able to activate this atypical way (47).

Findings showed that some complications due to aging could be treated by Bort therapy through preventing proteasome or inducing NF-κB pathways (48). Some studies claim that proteasome inhibition occurs at the end of this atypical NF-κB response.

Cullen *et al*. (2010) demonstrated that the effects of proteasome inhibition are age-dependent and play roles in NF-κB-mediated inflammatory gene transcription, geriatric diseases and hyperactive dysfunction (49).

## Conclusion

In our study, findings in Ovt group are consistent with the literature. Differently with the Cont group, in stromal cells of Ovt group, especially some cells of basal endometrium showed strong cytoplasmic and nuclear NF-κB positivity. However, the proteasome inhibition in the Bort group improved morphometric markers, especially in endometrium. In this case, Bort (proteasome inhibitor) could be considered as protective and curative agent on the endometrium. 

It is known that the ovariectomy has negative effects on the uterus. In this process, proteasome inhibition may have protective effects. In this study, the effects were shown by immunohistochemical and morphometric methods. At this point, further studies are needed to better understand the mechanisms of Bort effects on uterus of the ovariectomized rat.
